# Novel injectable polypeptide nanoparticle encapsulated siRNA targeting TGF‐β1 and COX‐2 for localized fat reduction I: Preclinical in vitro and animal models

**DOI:** 10.1111/jocd.16535

**Published:** 2024-08-21

**Authors:** Mark S. Nestor, John Hetzel, Nardin Awad, Vishnu Bhupalam, Patrick Lu, Michael Molyneaux

**Affiliations:** ^1^ Center for Clinical and Cosmetic Research Aventura Florida USA; ^2^ Department of Dermatology and Cutaneous Surgery University of Miami, Miller School of Medicine Miami Florida USA; ^3^ Sirnaomics Inc Gaithersburg Maryland USA

**Keywords:** adipose, COX‐2, fat, siRNA, TGF‐β1

## Abstract

**Background:**

Obesity and localized fat accumulation continue to drive the demand for minimally invasive body contouring technologies including injectable compounds for local fat reduction. siRNA offers a potential for an injectable to specifically target and silence genes involved in adipogenesis with minimal inflammatory side effects.

**Aims:**

This study evaluates the efficacy of STP705, an injectable containing siRNA encapsulated within histidine‐lysine polypeptide (HKP) nanoparticles targeting transforming growth factor β1 (TGF‐β1) and cyclooxygenase‐2 (COX‐2), crucial mediators in adipocyte differentiation and fat retention, using in vitro, porcine, and murine models.

**Methods:**

In vitro experiments on mouse preadipocytes and in vivo trials using Diet Induced Obese (DIO) mice and Yucatan minipigs were conducted to assess the gene silencing efficiency, tissue localization, pharmacodynamics, and safety profile of STP705.

**Results:**

STP705 effectively reduced the expression of TGF‐β1 and COX‐2, with a notable decrease in adipocyte volume and lipid content without adverse systemic effects. In DIO mice, the HKP‐siRNA complex demonstrated precise localization to injected adipose tissue, maintaining significant gene silencing, and detectable levels of siRNA for up to 14 days post‐administration. Similar results in minipigs showed a significant reduction in subcutaneous adipose tissue thickness.

**Conclusion:**

The results of these studies support the use of targeted siRNA therapy specifically targeting TGF‐β1 and COX‐2, for localized fat reduction, offering a potential minimally invasive alternative to current fat reduction methods.

## 1 INTRODUCTION

The demand for body contouring and fat reduction procedures is increasing markedly, a trend which is driven by many factors, such as the growing global emphasis on physical appearance and the surging prevalence of obesity.^1^ In concert with this developing demand, innovation over the past two decades has improved gold‐standard procedures, such as liposuction, while also opening frontiers for newer and now highly popular energy‐based and injectable fat‐reduction techniques. Deoxycholic acid (DCA), which is currently the only FDA‐approved agent for injection lipolysis, has shown efficacy in treating submental fat deposits, however, DCA treatment can involve significant patient downtime and is associated with side effects like local pain, redness, and inflammation.^2^ Given these issues, alternative options for fat reduction should be explored to optimize the balance between efficacy and side effects.

Recent advancements have highlighted the therapeutic potential of siRNA‐based treatments, but the nascent applications of this technology in fat reduction remain largely unexplored.^3^ Interventions based on siRNA suppression of pro‐adipogenic genes offer an alternative therapeutic approach for localized fat reduction that might better optimize efficacy and tolerability. Among the gene products implicated in stubborn fat, transforming growth factor β 1 (TGF‐β1), and cyclooxygenase 2 (COX2) are promising targets implicated in maintaining stubborn fat deposits. TGF‐β1 is a cytokine with pivotal roles in adipogenesis and fat distribution, while COX2 is an enzyme that promotes adipocyte differentiation and is highly expressed in metabolically active adipocytes.^4^, ^5^, ^6^ Local suppression of these factors using siRNA could disrupt adipocyte cellular processes, promote apoptosis, and yield a reduction in adipose tissue. This manuscript describes a series of preclinical in vitro and in vivo animal model investigations examining key aspects of pharmacokinetics, pharmacodynamics, safety, and efficacy of STP705 (Sirnaomics, Inc., Gaithersburg, MD), a novel injectable agent consisting of a histidine‐lysine polypeptide (HKP) vehicle complexed with siRNAs targeting TGF‐β1 and COX2 for the purpose of fat reduction.

## 2 PRECLINICAL EXPERIMENTAL METHODS AND RESULTS

### 2.1 Gene silencing efficacy of STP705 in mouse preadipocytes

#### 2.1.1 Hypothesis

- A novel HKP‐encapsulated dual‐targeted siRNA (STP705) will effectively reduce the expression of TGF‐β1 and COX‐2 in mouse preadipocytes.

#### 2.1.2 Methods

Mouse preadipocytes (3 T3‐L1) were transfected with STP705 (HKP/siTF1 + siCX2) or identically PNP‐complexed single‐target siRNA drugs for TGF‐β1 (HKP/siTF1) and COX‐2 (HKP/siCX2) in 200 nM, 100 nM, and 50 nM concentrations (calculated by molarity of siRNA). Cells transfected with 100 nM noncoding siRNA encapsulated in lipofectamine 2000 served as a negative control, and untreated cells served as a blank control. Following transfection, cells were cultured for 20–24 h, RNA was extracted, and target gene expression levels were quantified using RT‐PCR.

#### 2.1.3 Results

Both target genes were effectively silenced by all transfection levels of their respective single‐target drugs and STP705. Compared with the blank control, HKP/siTF1 and STP705 silenced the mRNA expression of TGF‐β1 by 46%–60%, and each drug showed a dose‐dependent effect (Figure 1A). STP705 displayed a mild synergistic effect on gene silencing as compared to HKP/siTF1—for example, 100 nM STP705 (comprised of 50 nM siTF1 and 50 nM siCX2) silenced 51% of expression as compared to 50 nM siTF1, which silenced 46%. HKP/siCX2 and STP705 silenced the mRNA expression of COX2 by 34%–48% in a dose‐dependent manner as compared to the blank control (Figure 1B). HKP/siCX2 and STP705 appeared to be equally effective at silencing COX‐2 on a cellular level.

**FIGURE 1:**
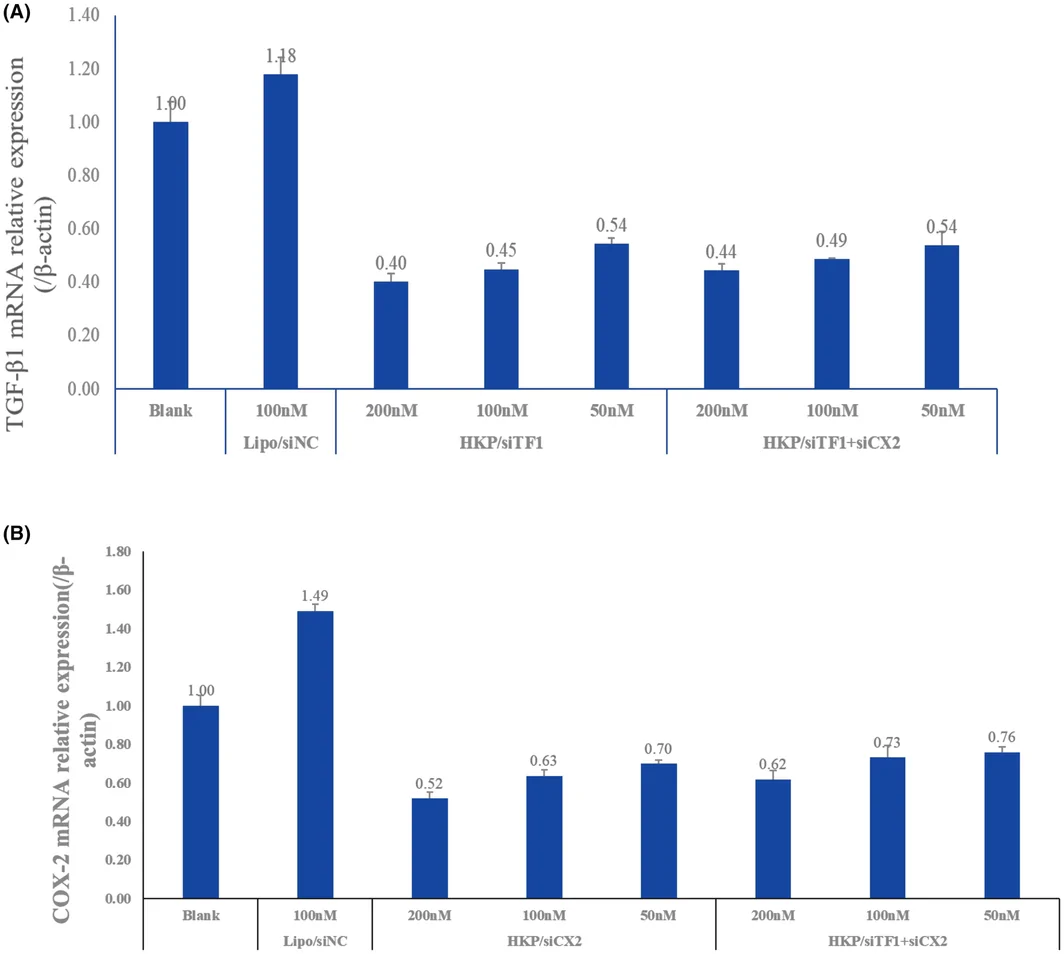
(A) Both target genes were effectively silenced by all transfection levels of both single‐target drugs and STP705. HKP/siTF1 and STP705 silenced the mRNA expression of TGF‐β1 by 46%–60%, and each drug showed a dose‐dependent effect. (B) HKP/siCX2 and STP705 appeared to be equally effective at silencing COX‐2 on a cellular level.

### 2.2 Conclusion

Single and dual‐target PNP‐complexed siRNA agents were effective at eliciting a significant and dose‐dependent reduction in the expression of TGF‐β1 and/or COX‐2 in mouse preadipocytes. Given this success in modulating the proposed targets in vitro, there was sufficient evidence for subsequent in vivo investigations exploring the tissue localization of the siRNA agent following injection.

### 2.3 Retention of PNP‐complexed siRNA after inguinal fat injection in DIO mice

#### 2.3.1 Hypothesis

- HKP‐encapsulated siRNA agents will be retained locally following injection into the inguinal fat of DIO mice.

#### 2.3.2 Methods

Thirty diet‐induced obesity (DIO) mice were administered a single dose of Cy3 fluorescent dye‐tagged HKP/siTF1 (9 μg in a volume of 90 μL) into the right inguinal fat in equal 30 μL aliquots at three injection points. Following injection, three random mice were selected for anesthetized live imaging and assessment of Cy3 fluorescence intensity at 4 h, 24 h, 48 h, 72 h, 120 h, 168 h, 216 h, 264 h, 312 h, and 336 h time points (Figure 2B).

**FIGURE 2:**
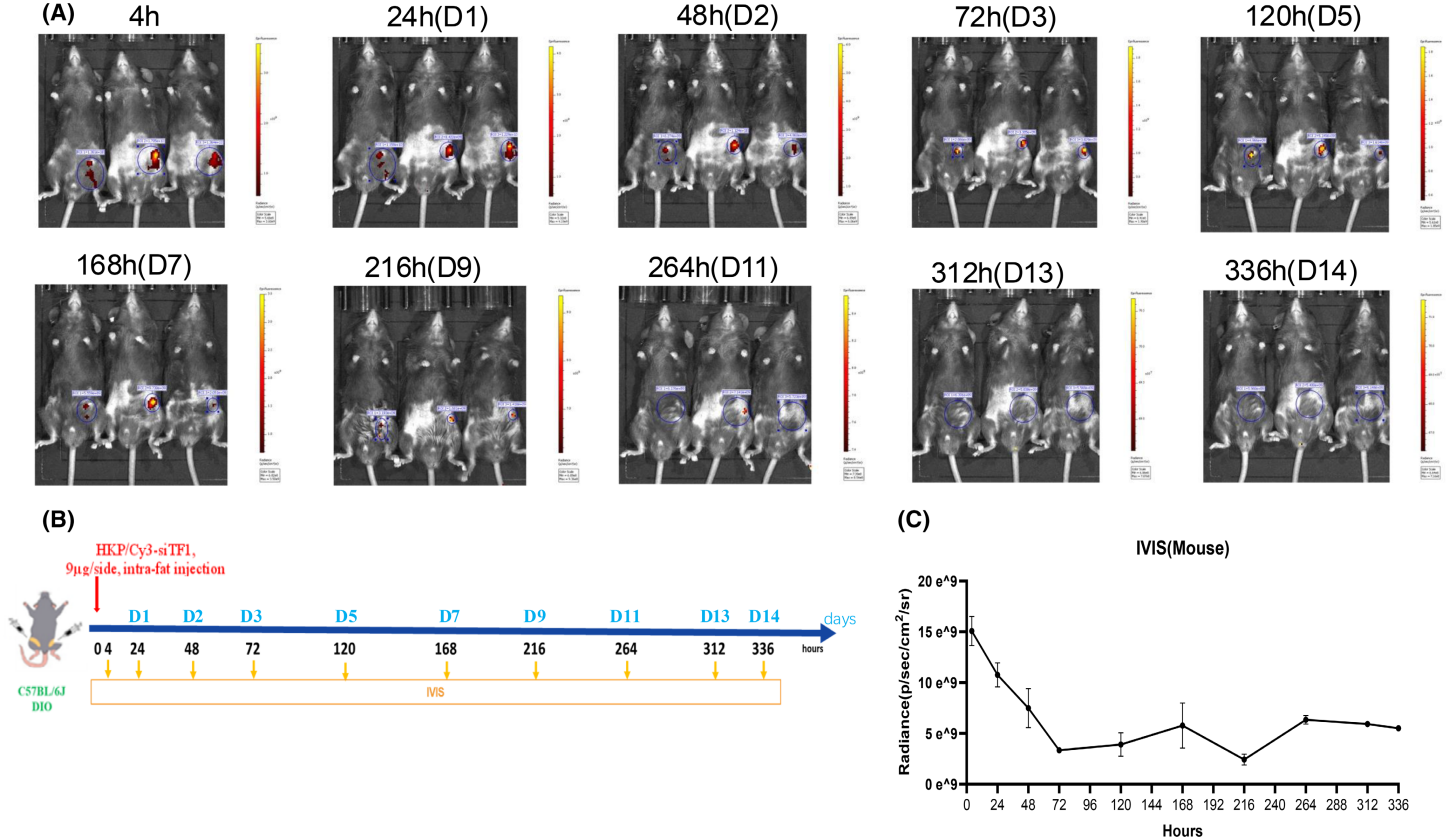
(A). After a local injection of HKP/Cy3 siTF1 into inguinal fat, fluorescence signals could be clearly detected at the injection site. (B) Thirty diet‐induced obesity (DIO) mice were administered a single dose of Cy3 fluorescent dye‐tagged HKP/siTF1 (9 μg in a volume of 90 μL) into the right inguinal fat in equal 30 μL aliquots at three injection points. Following injection, 3 random mice were selected for anesthetized live imaging and assessment of Cy3 fluorescence intensity at 4 h, 24 h, 48 h, 72 h, 120 h, 168 , 216 h, 264 h, 312 h, and 336 h time points. (C) The fluorescence signals gradually weakened and reached a relatively stable state after 72 h but could still be observed at 14 days after administration.

### 2.4 Results

Fluorescence signals were clearly detected at the inguinal injection site following a single Cy3‐tagged HKP/siTF1 injection (Figure 2A). The fluorescence signal diminished gradually over 72 h and then plateaued at a detectable intensity for at least 14 days postinjection (Figure 2C).

### 2.5 Conclusion

As demonstrated by fluorescence imaging, PNP‐complexed siRNA agents were retained within the inguinal tissues following local injection. This suggested that the novel PNP vehicle was effective at promoting the localization of the transported siRNA agent into the targeted tissue, thereby warranting additional in vivo investigation of agent efficacy in modulating gene expression within adipose tissue.

### 2.6 Tissue distribution and gene silencing activity of PNP‐complexed siRNA after inguinal fat injection in DIO mice

#### 2.6.1 Hypothesis

- HKP‐encapsulated siRNA agents will be stable and effective at locally reducing target gene expression following injection into the inguinal fat of DIO mice.
- HKP‐encapsulated siRNA agents will be absorbed and retained locally and will not migrate into other tissues following injection into the inguinal fat of DIO mice.

### 2.7 Methods

Thirty DIO mice were divided into seven groups. Group 1 consisted of six untreated mice and served as a blank control. Groups 2–7 (*n* = 4 per group) received identical treatments consisting of Cy3‐tagged HKP/siTF1 injected bilaterally into the inguinal fat (9 μg/90 μL per side) at three injection points (per side) in equal aliquots of 30 μL. Groups 2–7 correspond sequentially to postinjection time points of 4 h, 24 h, 72 h, 120 h, 168 h, and 240 h. At each time point following treatment administration, mice in the corresponding group were anesthetized and harvested of inguinal fat, lymph nodes, brain, heart, liver, spleen, lungs, and kidneys, which were then imaged to evaluate the tissue distribution of Cy3‐tagged HKP/siTF1. During tissue imaging, corresponding tissues from one mouse in the blank group were used to adjust the fluorescence baseline. Additionally, inguinal fat was processed for gene silencing and siRNA residue detection at each time point. Total RNA was extracted from the inguinal fat using the Trizol method, and target gene expression was quantified using RT‐PCR. The content of siRNA antisense chains in the inguinal fat was determined using stem‐loop RT‐QPCR.

### 2.8 Results

Fluorescence signals from Cy3‐tagged HKP/siTF1 were detectable at all time points for the adipose tissue collected from the injection site; the signals decreased gradually over time (Figure 3A). However, no Cy3 fluorescence signals were detected in any of the other organ tissues. Large amounts of siRNA residue were detected in the adipose tissue, and the quantity peaked at about 46% of the administered dose within 24 h of injection (Figure 3B). At 240 h postinjection, about 6% of the dosage was still detected within the inguinal fat. The expression of TGF‐β1 mRNA in the adipose tissue at the injection site was significantly suppressed following treatment. Compared to the blank, TGF‐β1 mRNA expression was reduced by 30% after 4 h (*p* < 0.05), 63% at 24 h (*p* < 0.001), and 90% after 120 h (*p* < 0.0001) (Figure 3C). Though TGF‐β1 mRNA expression rebounded somewhat at 240 h, it continued to be significantly suppressed by 57% compared to the control (*p* < 0.05).

**FIGURE 3:**
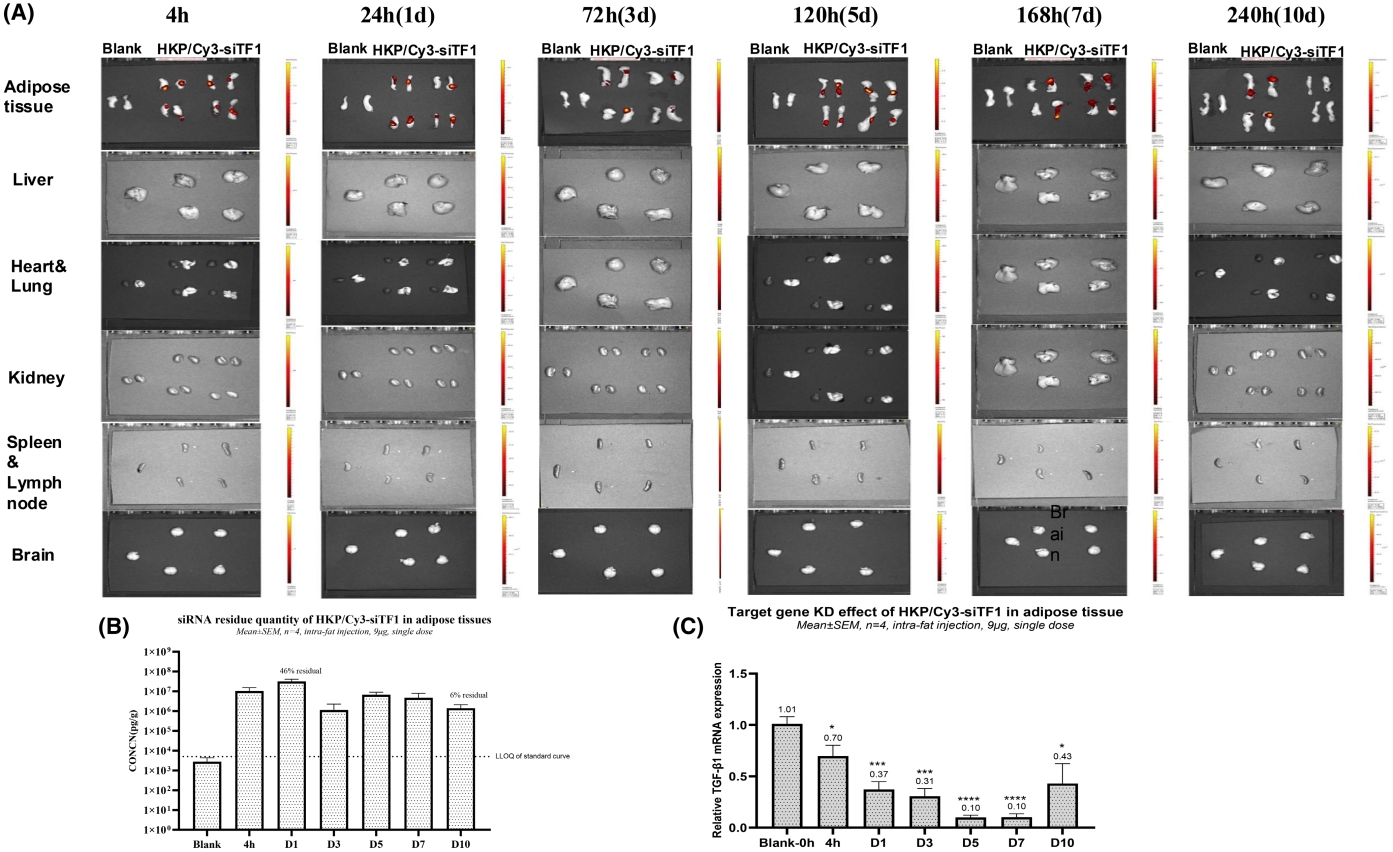
(A) Tissue imaging was performed at 4 h, 24 h (1D), 72 h (3D), 120 h (5D), 168 h (7D), and 240 h (10D) after an injection of HKP/Cy3 siTF1 into inguinal fat of DIO mice. Significant fluorescence signals could be detected in the adipose tissue at the injection site and gradually decreased over time. No fluorescence positive signals were detected in other organ tissues. (B) Within 1 day of administration, over 107 pg/g (about 46% of the dosage) siRNA residue was still detected in adipose tissue. After 10 days of administration, there was 106 pg/g residue (about 6% of the dosage). (C) TGF‐β1 mRNA expression in adipose tissue at the injection site was significantly knocked down after HKP/Cy3‐siTF1 treatment. Expression in adipose tissue was silenced by 30% at 4 h, 63% at 1D, 69% at 3D, 90% at 5D and 7D after administration. TGF‐β1 mRNA expression had rebounded at 10D after administration, but there was still a 57% knockdown. *p < 0.05; ***p<0.001; ****p < 0.0001 all versus Blank.

### 2.9 Conclusion

This experiment verified that the targeted gene suppression achieved with PNP‐siRNA agents during the previous in vitro investigation remains significant in vivo. It also provided further evidence that the PNP vehicle achieves excellent localization to the targeted adipose tissue without uptake into other organs. These results prompted an experiment to provide more granular detail on the siRNA agent's localization to specific cell populations within inguinal fat deposits.

### 2.10 Cell type distribution in the inguinal fat of DIO mice after PNP‐siRNA injection

#### 2.10.1 Hypothesis

- HKP‐encapsulated siRNA agents will exhibit local uptake into adipocytes and their precursors following injection into the inguinal fat of DIO mice.
- HKP‐encapsulated siRNA agents will not adversely influence the population proportions of off‐target cell types in the short term.

### 2.11 Methods

Six DIO mice were injected with Cy3‐tagged HKP/siTF1 in the left inguinal fat. The injection dose was 10 μg/100 μL divided into equal 25 μL aliquots at four injection points. To serve as a control, the same mice received injections of an equal volume of saline into the right inguinal fat. After 48 h, the mice were anesthetized and harvested for inguinal fat (Figure 4A). The inguinal fat was processed into a single‐cell suspension, and flow cytometry was used to quantify the presence of several cell types, such as macrophages, adipocyte stem and precursor cells (ASPCs), and mature adipocytes. The proportion of fluorescence signal‐positive cells was also determined for each cell type as compared to controls.

**FIGURE 4:**
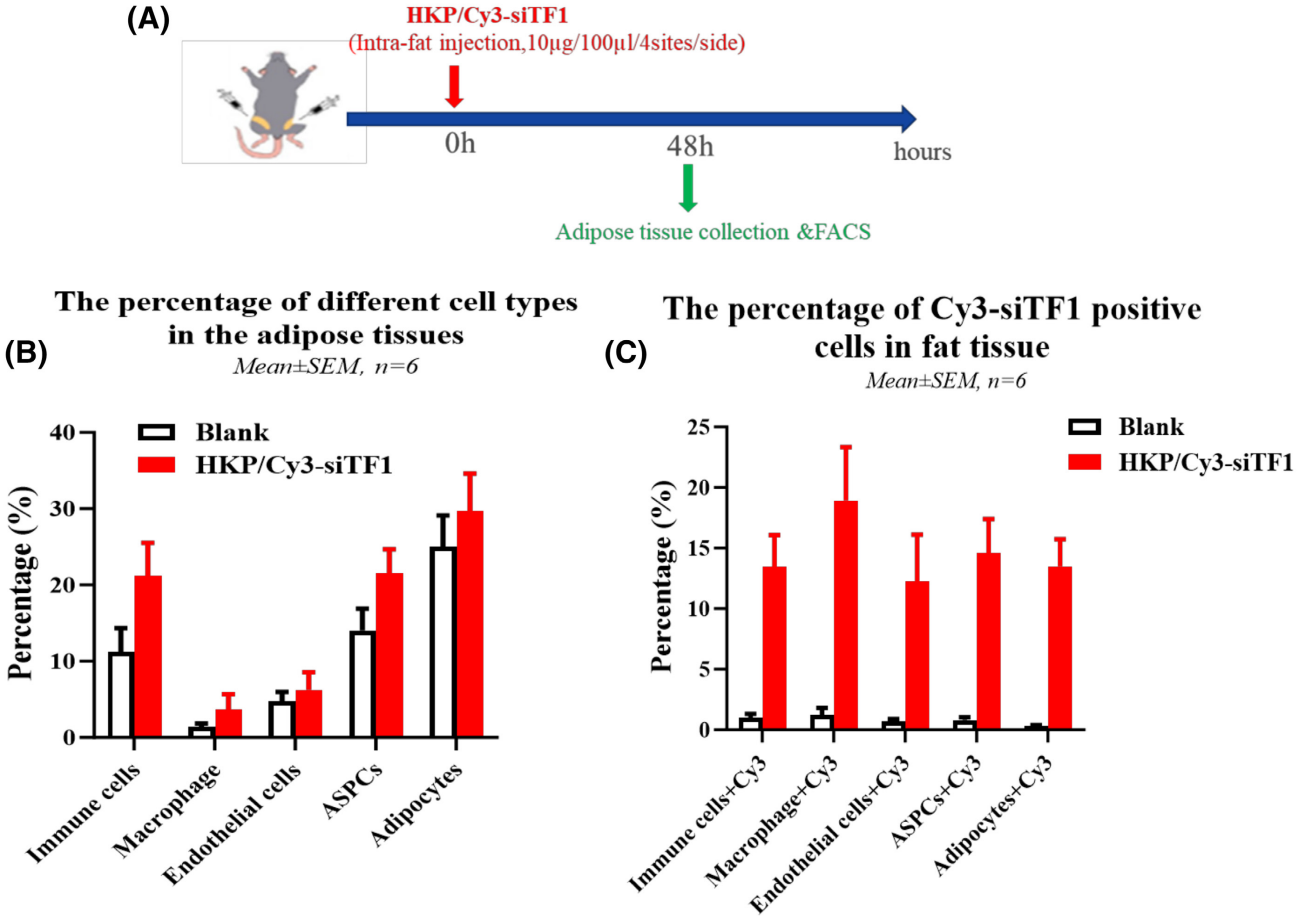
(A) Six DIO mice were given HKP/Cy3‐siTF1 injections into the left inguinal fat and saline injections into the right inguinal fat. Inguinal fat was collected at 48 h after administration to investigate the cell type distribution. (B) There was no significant change in the number of major cell types in adipose tissue treated with HKP/Cy3‐siTF. Adipocytes and adipose stem and progenitor cells (ASPC) were the main cells in adipose tissue, accounting for over 50% of the total number of cells. (C) Cy3 fluorescence signals were detected in approximately 15% of adipocytes or adipose stem or precursor cells (ASPC), suggesting that HKP/Cy3‐siTF can enter adipocytes and ASPC.

### 2.12 Results

Compared with the control side injected with saline, there was no significant change in the number or proportion of major cell types in adipose tissues treated with an injection of HKP/siTF1 (Figure 4B). Mature adipocytes and ASPCs comprised the majority (>50%) of cells in the harvested tissue. Fluorescence signals for Cy3 were observed to varying extents in all cell types on the treatment side, including mature adipocytes and ASPCs, for which ~15% of cells exhibited signal positivity at 48 h postinjection (Figure 4C).

### 2.13 Conclusion

The PNP‐siRNA vehicle was effective at localizing to the intended cell types (mature adipocytes and ASPCs) with variable uptake into off‐target cell types. There were no short‐term changes in the proportions of key cell types. Given the accumulated evidence indicating that PNP‐siRNA agents are effective at localizing to the target tissue and modulating the intended gene expression profiles, a follow‐up investigation was warranted to assess key pharmacodynamic aspects and preliminary efficacy measures for STP705.

### 2.14 Pharmacodynamics of STP705 administered for focal fat reduction in DIO mice

#### 2.14.1 Hypothesis

- A novel HKP‐encapsulated siRNA agent (STP705) targeting TGF‐β1 and COX‐2 will effectively reduce local fat when injected into the inguinal fat of DIO mice.
- STP705 will reduce the content of triglycerides and lipids following injection into the inguinal fat of DIO mice.
- STP705 will increase adipocyte apoptosis following injection into the inguinal fat of DIO mice.
- STP705 will positively influence the expression of key factors in fat metabolism (CPT1) following injection into the inguinal fat of DIO mice.

### 2.15 Methods

A total of 42 DIO mice were randomly divided into 6 groups (*n* = 7 per group). Group 1 = Model (DIO), Group 2 = STP705 (3 μg), Group 3 = STP705 (10 μg), Group 4 = STP705 (30 μg), Group 5 = siNC (30 μg) (NC; negative control), and Group 6 = DCA (2000 μg). Corresponding doses of STP705, DCA, siNC, or equivalent volumes of normal saline (NS) were injected into the left and right inguinal fat in four sites per side. Mice were administered injections once weekly for a total of three injections (with injection three occurring on the 14th day following initial treatment). Mice were weighed prior to treatment and then every 3 days. On the 21st day after the initial injection, mice were anesthetized and harvested for inguinal fat, which was weighed to evaluate fat reduction efficacy. Samples of fat tissue were fixed in formalin, frozen, embedded, and sectioned at a 10 μm thickness. Oil Red O and TUNEL stains were performed and then analyzed by a blinded observer using Image Pro Plus software. Additionally, total RNA was isolated from a sample of inguinal fat using the Trizol method, and the mRNA expression of carnitine palmitoyl transferase 1 (CPT1) was quantified using RT‐PCR.

### 2.16 Results

In comparison to the gradual upward trend in weight for mice in the model group, the mice treated with STP705 displayed a relatively stable weight (Figure 5B). Treatment with injections of high‐dose STP705 (30 μg) significantly decreased the weight of inguinal fat when compared to the model (*p* < 0.01) and the negative control (*p* < 0.05) (Figure 5C). In general, STP705 demonstrated a weight reduction effect that was at least equal to that of DCA (2000 μg). Additionally, compared to the model group, intra‐fat injection with STP705 caused a dose‐responsive reduction of up to 36% (30 μg) in the content of neutral triglycerides and lipids in adipose tissue, whereas DCA achieved only approximately 24% reduction (Figure 5D,E). Intra‐fat injection of STP705 (10 μg and 30 μg) also caused a significant dose‐dependent increase in the expression of CPT1 by up to 59% as compared to the control; DCA treatment showed no change in this factor (Figure 5F). Lastly, STP705 (3 μg, 10 μg, 30 μg) caused a dose‐responsive increase in the number of apoptotic cells within the inguinal fat, and STP705 30 μg showed greater efficacy for this than DCA 2000 μg (Figure 5G,H).

**FIGURE 5:**
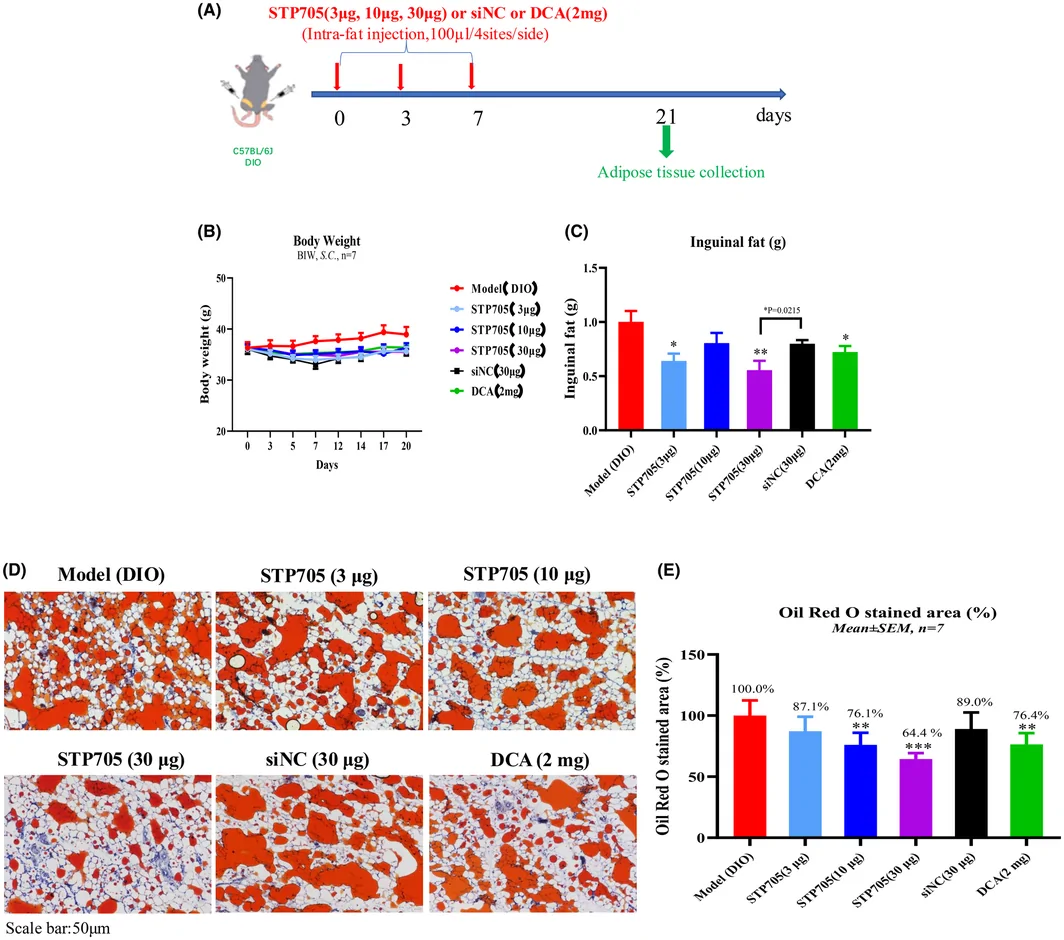
(A) DIO mice were randomly divided into 6 groups and injected three times into the inguinal fat. Inguinal fat was collected and weighed to evaluate local fat reduction 21 days after the first administration. (B) The body weight of the treated mice was relatively stable compared to a slight upward trend in the model group. (C) Intra‐fat injection of STP705 at a high dose (30 μg) significantly reduced the fat weight at the injection site by about 50%. The overall effect of STP705 showed a dose–response effect which was better than that of DCA (2 mg) administration (about 25% weight loss). (D, E) Intra‐fat injection of STP705 (10 μg and 30 μg) significantly reduced the neutral triglycerides and lipids of adipose tissue at the injection site by 24% and 36% respectively. There was a dose–response effect which was better than that of DCA (2 mg) administration (approximately 24%).

### 2.17 Conclusion

Intra‐fat injections of STP705 were demonstrated to be comparably effective to DCA at reducing the weight of inguinal fat. At higher doses, STP705 was more effective at decreasing lipid content, promoting the expression of CPT1 (a key enzyme in fat metabolism), and increasing apoptosis within inguinal fat as compared to DCA. These results substantiated the efficacy of targeted gene suppression for the purpose of fat reduction and led naturally to the exploration of STP705's efficacy and preliminary clinical dosing parameters in a separate animal model.

### 2.18 Efficacy, safety, and dosing parameters of STP705 intra‐fat injection for focal fat reduction in Yucatan minipigs

#### 2.18.1 Hypothesis

- STP705 will reduce local fat when injected into Yucatan minipigs.

#### 2.18.2 Methods

Male Yucatan minipigs (*n*3) were assigned to receive treatment 1, 2, or 3 times. Treatment consisted of a set of injections of varying doses of STP705 (120 μg, 200 μg, 400 μg, 640 μg), STP705‐2 (120 μg and 200 μg) and a set dose of DCA (2000 μg) administered by injection into the subdermal adipose tissue of the back at three separate designated sites. Treatments were administered 14 days apart, with day 0 being set as the first treatment day. Sequential treatments were administered at the same sites (for each dose) as the first treatment. Minipig 1001 received treatment on day 0; Minipig 2001 received treatment on days 0 and 14; Minipig 3001 received treatment on days 0, 14, and 21. Pre‐dosing biopsies were collected. Animals were euthanized on day 56 and biopsies were taken. An independent histopathology lab analyzed each biopsy for effects on subcutaneous adipose, dermal, and epidermal tissues.

Skin samples were routinely processed during histopathologic evaluation. Samples were bisected at the injection site and embedded cut side down, both pieces in a single block. One slide per block was sectioned and stained with hematoxylin and eosin (H&E). Glass slides were evaluated by a board‐certified veterinary pathologist using light microscopy. Histologic lesions of dermal inflammation, serocellular surface crust (keratin and exudate accumulation on surface), subcuticular inflammation and deep dermal/subcuticular fibrosis/fibroplasia were graded for severity based on % area affected according to the following criteria: (0 = absent; 0.5 = 1%–4%; 1 = 5%–10%; 2 = 11%–25%; 3 = 26%–50%; 4 = 51%–75%; 5 = >75%). An ocular micrometer was used to measure the thickness in a single location that best represented the overall epidermal morphology. To determine if the treatments reduced the subcuticular adipose later, subcuticular adipose thickness (μm) was measured in 10 sites across each sample. Slides were also scanned using an Aperio AT2 slide scanner for the creation of whole slide images, and additional measurements were performed using Aperio ImageScope software.

### 2.19 Results

The untreated control sites from Minipigs 2001 and 3001 exhibited inflammation likely due to nematode larvae migration and possible site excision issues. Nematode larvae were identified in one of three sites in Minipig 2001. These control sites showed mild to severe inflammation, fibrosis, and granulomatous inflammation, whereas the control site in Minipig 1001 had minimal inflammation and no fibrosis, thus the control site from Minipig 1001 was used as the control for the test article treatments in all three animals.

Histologically, the response to DCA injection primarily elicited granulomatous inflammation, whereas STP705 induced both granulomatous inflammation and distinctive mineralization patterns. These responses differed from each other and from the response to nematode larvae, aiding in distinguishing the sources of inflammation.

Pre‐dose biopsies showed minimal inflammation of the dermis without other lesions. Since the animals were still growing during the study the pre dose adipose layer was thinner than the control biopsies taken at the end of the experiment. In aggregate, STP705 and STP705‐2 treatments at all dosing levels resulted in significant reductions in subcuticular adipose thickness compared to control and DCA (Figure 6), with then histopathology showing stark reduction in the adipose tissue layer and fibrosis (Figure 7).

**FIGURE 6:**
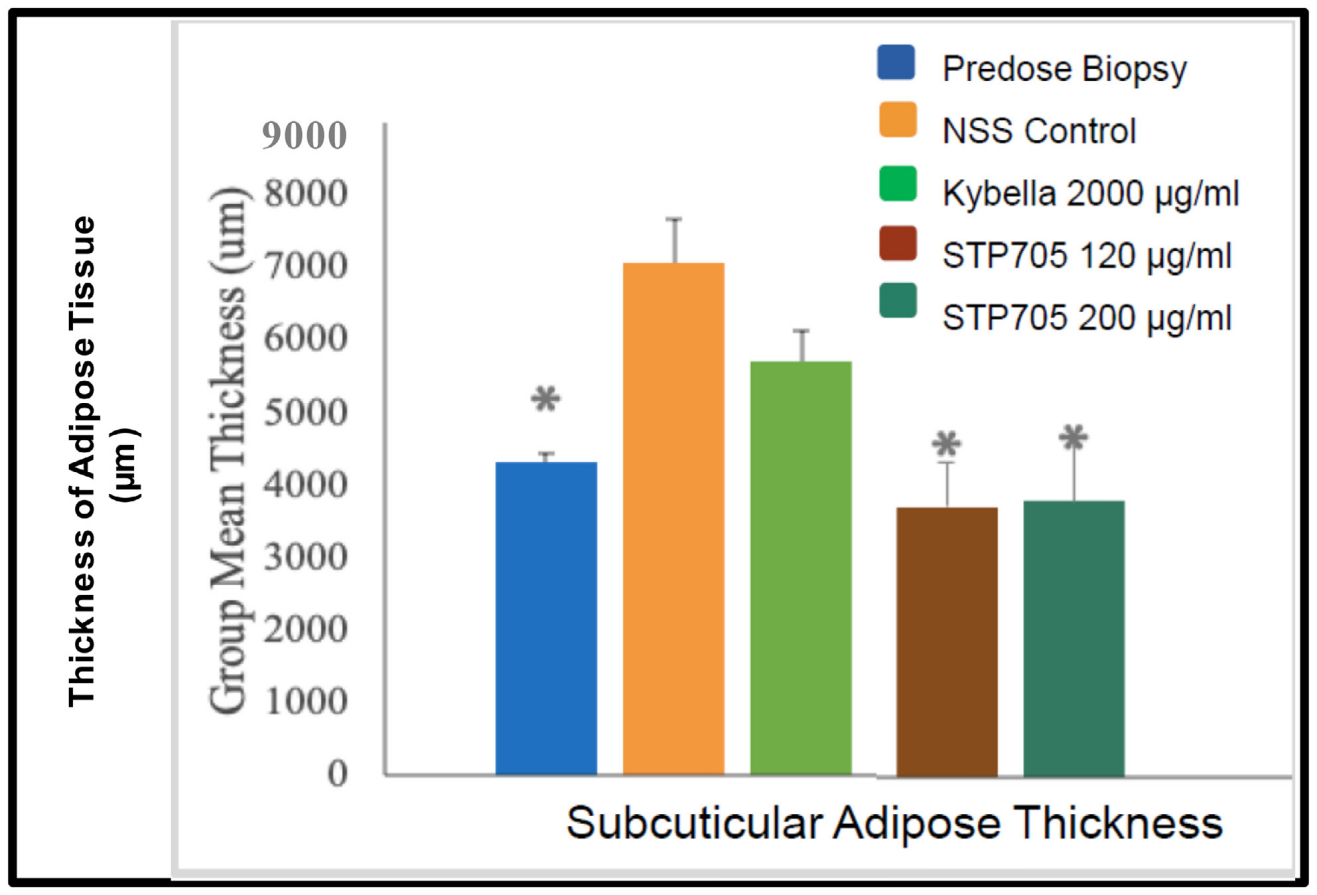
Graphical comparison of the efficacy of DCA and STP705 in reduction of adipose tissue thickness.

**FIGURE 7:**
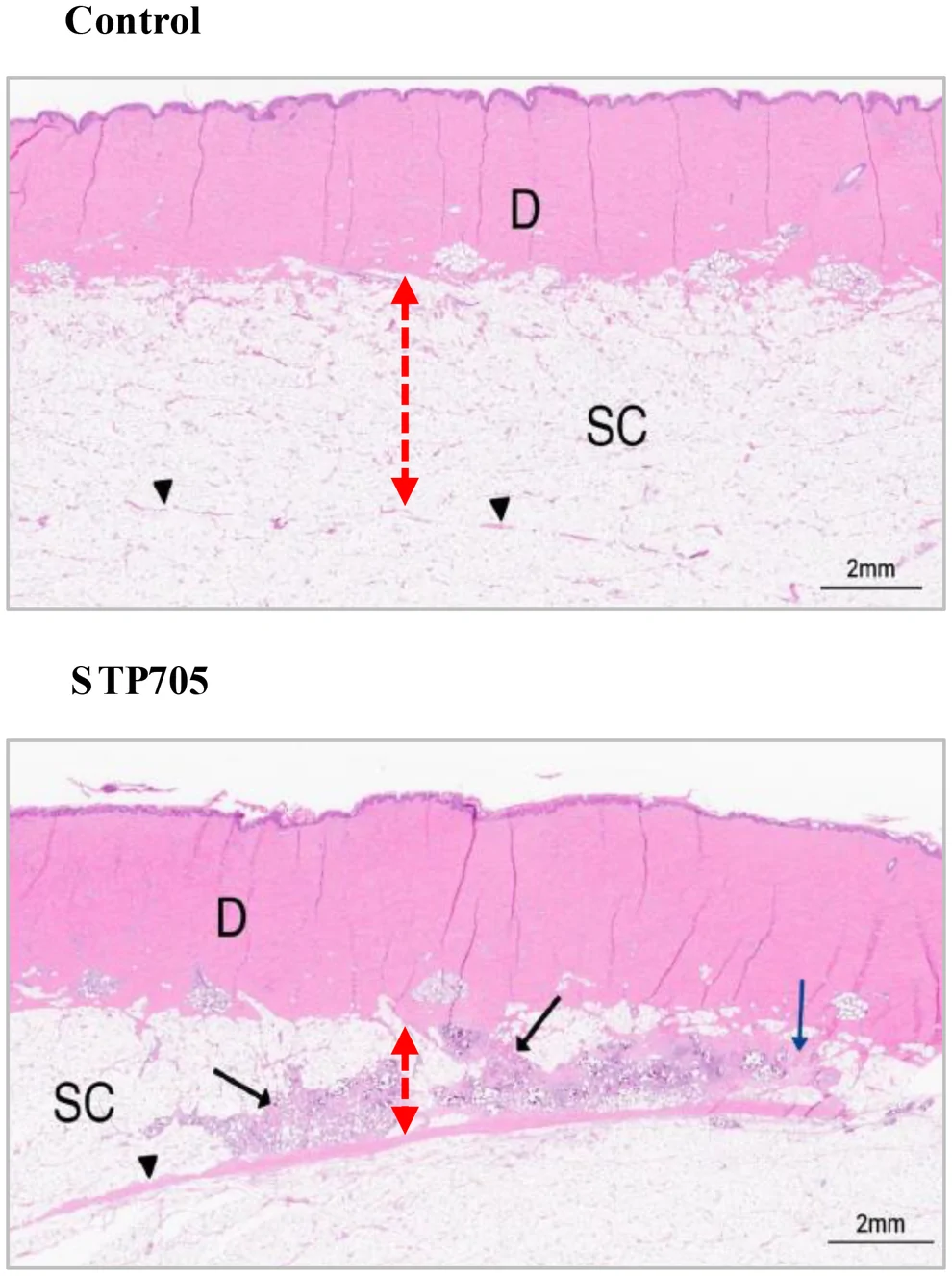
Histological comparison of subcutaneous thickness in a control versus STP705‐treated animals.

### 2.20 Conclusion

Comparison of pre‐dose and nontreated control samples to treated samples with both DCA and STP705 showed that both DCA and STP705 treatments caused reductions in the thickness of the adipose tissue with STP705 showing significantly greater reduction. The inflammatory response to both treatments involved macrophage infiltration, with STP705 demonstrating characteristic mineralization, aiding in differentiation.

## 3 DISCUSSION

Broadly speaking, localized fat reduction is achievable through three distinct mechanisms: surgical extraction of adipose tissue (i.e., liposuction), direct damage and destruction of adipocytes, and exploitation of intrinsic cell signaling pathways. Of these, the latter two are the principal mechanisms for all noninvasive fat reduction procedures, such as cryolipolysis and deoxycholate injection. In both energy‐based and chemical lipolytic procedures, the primary goal is to selectively cause sufficient adipocyte damage (often to the sensitive lipid membrane) so as to impair integral cellular functions and cause rapid cell death via necrosis or apoptosis. As a secondary effect of elevated adipocyte stress and disorganized cell death (i.e., necrosis), this process also elicits significant inflammatory signaling that has been linked to further adipocyte apoptosis. Adipocytes that are sufficiently dysfunctional or have undergone cell death are subsequently recycled by tissue macrophages, and excess fatty acids from this process are redistributed to other body compartments.^7^

Advancements in the understanding of adipose tissue biology have highlighted certain cellular factors, such as enzymes and cytokines, as having an important role in maintaining stubborn fat deposits.^8^ Concurrently, a broad scientific movement has placed greater focus on the role of gene expression in disease pathogenesis and driven major developments in agents targeting specific gene products, such as therapies based on small‐interfering ribonucleic acid (siRNA).^3^, ^9^ During the process of RNA interference, siRNA strands bind to and degrade complementary mRNA sequences, thereby inhibiting the translation of their respective proteins and effectively achieving gene suppression.^10^ Given that each siRNA is inherently specific to its gene targets, therapeutic agents based on siRNA have great potential as flexible and precise treatments for a broad range of issues. In the case of localized adiposity, siRNA disruption of key cell signaling factors may safely and selectively impair adipocytes such that they undergo apoptosis and are removed from the location of concern.

Though there were initial challenges in delivering siRNA to target cells due to its anionic charge and propensity for rapid degradation, the clinical viability of siRNA drugs has been enhanced by innovations in delivery systems that improve stability, reduce immune responses, and facilitate efficient uptake at targeted cells.^3^ Among the solutions devised to address shortfalls in drug delivery, polypeptide nanoparticles (PNPs) have emerged as a promising vehicle for siRNA agents. PNPs encapsulate the siRNA, protecting it from degradation in the systemic circulation and enhancing its delivery by allowing for passage through lipid bilayers. Through surface modifications and structure optimization, the design of PNP vehicles can be tailored further to improve their affinity for specific cells, such as adipocytes. Together, these alterations improve siRNA drug efficacy and decrease off‐target effects, thereby facilitating a lower effective dose requirement and better safety profile.^3^, ^11^

The results of this series of experiments indicate that the novel siRNA agent STP705 effectively silences TGF‐β1 and COX2 in preadipocytes by decreasing mRNA expression (Figure 1A,B). This gene‐silencing effect was marginally dose‐dependent and synergistic in nature compared to HKP/siTF1, suggesting a potential for precise therapeutic control (Figure 1). Particularly noteworthy was the sustained presence of siRNA in adipose tissue, as evidenced by fluorescence imaging. After a single local injection of HKP/Cy3‐siTF1 into inguinal fat, fluorescence signals could be clearly detected at the injection site. The fluorescence signals gradually weakened and reached a relatively stable state after 72 h but could still be observed at 14 days after administration, indicating that HKP/Cy3‐siTF1 could be retained in inguinal fat for at least 14 days (Figure 2C). In a separate study, siRNA residue was still detected in adipose tissue 10 days following the initial injection, and TGF‐β1 mRNA expression at the injection site was significantly decreased, again confirming the retention of the siRNA within the adipose tissue, as well as the gene silencing mechanism of siRNA (Figure 3B,C). This speaks to the feasibility of long‐term therapeutic effects. These findings align with the current understanding of RNA interference mechanisms, where the specificity and efficiency of gene silencing are crucial for therapeutic success.

Tissue distribution of HKP/Cy3‐siTF1 in various tissues and organs of DIO mice was evaluated after a single injection of HKP/Cy3‐siTF1 into the inguinal fat. No fluorescence‐positive signals were detected in other organ tissues (including brain, heart, liver, spleen, lungs, kidneys, and lymph nodes), and was found solely in adipose tissue, indicating that HKP/Cy3 siTF1 does not enter other tissues or organs (Figure 3A). The results demonstrated minimal off‐target effects and specific uptake by targeted cell types, which is paramount for minimizing potential systemic effects. These findings contribute to the growing body of evidence supporting the safety of siRNA therapies, particularly when complexed with nanoparticles that enhance target specificity.

DCA is a secondary bile acid produced in the intestines and stored in the gallbladder that assists in the breakdown and absorption of dietary fats by the gastrointestinal tract through emulsification and solubilization. Synthetic DCA was approved by the FDA for submental fat reduction in 2015 and acts by disrupting the cell membrane of adipocytes which stimulates a local inflammatory response with fibroblast recruitment and neocollagenesis, resulting in local adipocytolysis.^2^, ^12^ DCA injection is, however, frequently associated with significant adverse events, including pain, bruising, and numbness, as well as more serious events, including alopecia, nerve injury, and skin necrosis.^2^ When compared with DCA, the effect of STP705 was significantly better at decreasing fat weight and reducing levels of neutral triglycerides and lipids in adipose tissue (Figure 5C–E). Likewise, STP705 was superior to DCA at increasing adipose tissue expression of CPT1 (a rate‐limiting enzyme in the β‐oxidation of long‐chain fatty acids), and it caused a greater increase in adipocyte apoptosis than DCA (Figure 5F–H). The effects of STP705 were dose‐dependent and clearly superior to DCA. The dose‐dependent reduction observed in these studies underlines the potential of siRNA in localized fat reduction, offering a more targeted and possibly safer option for patients.

The cell‐level effects of STP705 observed in these studies, such as reductions in triglycerides and increased expression of a key fat metabolism enzyme, find resonance in a growing body of literature emphasizing the roles of TGF‐β1 and COX‐2 in adipose tissue. TGF‐β1 is a multifunctional cytokine that plays a vital role in many cellular processes, including cell growth, differentiation, apoptosis, immune regulation, and wound healing. TGF‐β1's role in adipocyte function and adiposity is equally significant, influencing adipogenesis and fat distribution. Enhanced amounts of TGF‐β1 mRNA and protein have been found in genetically obese mice compared to lean mice, with similar findings seen in obese humans compared to lean humans.^4^, ^8^ TGF‐β1 has been found to be strongly expressed by human adipose tissue and its antigen level increased in proportion with the degree of obesity.^5^ TGF‐β1 has also been shown to increase preadipocyte cell proliferation in many species, thus its increased expression in adipose tissue may increase adipocyte precursor cell proliferation.^4^ COX2 is an enzyme known for its role in inflammation and pain pathways. Beyond these functions, COX2 has a notable influence on adipose tissue biology. It has been shown to increase adipocyte differentiation and plays a role in the metabolic activity of adipocytes where it has been shown to be highly expressed.^6^, ^13^ By the same token, the inhibition or reduction of COX2 has been shown to cause a decrease in the expression of adipocyte‐specific genes through an inhibition of PPARγ mRNA expression. PPARγ is a characteristic of the adipocyte phenotype, and its expression is reduced when adipocyte differentiation is inhibited.^13^ The regulation of COX2 expression in adipose tissue can impact the process of adipogenesis and the overall function of fat cells, linking it to obesity as well as obesity‐associated complications.^6^ Given its role in both inflammation and adipocyte biology, COX2 presents itself as a potential therapeutic target in strategies aimed at enhancing fat reduction and improving the outcomes of procedures that involve remodeling or reduction of adipose tissue.

Our findings demonstrate siRNA's potential for precisely modulating pathogenically implicated gene targets. This is particularly significant when considering the limitations of current pharmacological agents, which often lack specificity and can lead to significant side effects. siRNA‐based therapy, however, functions through binding to a distinct mRNA target sequence, leading to gene suppression. The use of a polypeptide nanoparticle delivery system in these studies further aligns with recent advances in nanotechnology for drug delivery, enhancing target specificity and drug stability, as evidenced in the works by Han et al. (2014) and Rassu et al. (2017), who reported similar successes in targeted gene therapy using nanoparticle systems for cancer and Alzheimer therapy.^14^, ^15^ Han et al. prepared nanoparticles which were composed with galactose‐modified trimethylchitosan‐cysteine and loaded with siRNA that inhibit the expression of vascular endothelial growth factor (VEGF). The nanoparticles accumulated at the tumor site and effectively inhibited cell proliferation and tumor growth.^14^ The study by Rassu et al. showed therapeutic effects in Alzheimer's disease using BACE1 siRNA and rabies virus glycoprotein which were co‐loaded into nanoparticles.^15^ There are several types of nanoparticles that may be used as delivery systems, each of which is able to target a specific type of cell or tissue, leading to more specific, targeted delivery of siRNA, especially when conjugated to a ligand that allows for receptor‐mediated endocytosis.^16^

The clinical implications of these experiments are significant. The application of siRNA in nonsurgical fat reduction opens new avenues in cosmetic and other treatments. The observed efficacy in localized fat reduction presents a promising alternative to current treatments like liposuction or DCA injections, which can be invasive or associated with significant side effects. The specificity and safety profile of our siRNA therapy, as evidenced in our studies, could make it an appealing option for patients seeking minimally invasive localized fat reduction.

While these findings are promising, it is essential to acknowledge the limitations of translating results from animal models to human applications. Clinical trials should aim to establish the safety and efficacy of siRNA therapies in human subjects, considering variables such as individual genetic differences, long‐term gene expression patterns, and potential immune responses. Studies exploring the optimization of delivery systems and dosing regimens will also be important in enhancing the clinical applicability of siRNA‐based treatments.

This series of experiments represents a significant step forward in the field of RNA‐based therapeutics. By demonstrating the potential of siRNA in targeting key genes involved in adiposity, the preclinical studies pave the way for new therapeutic strategies that could revolutionize fat reduction treatments by laying a foundation for clinical trials.

## AUTHOR CONTRIBUTIONS

All authors met journal contribution guidelines.

## FUNDING INFORMATION

Sirnaomics, Inc., Gaithersburg, MD.

## CONFLICT OF INTEREST STATEMENT

Dr. Nestor is a consultant for Sirnaomics, Dr's Lu and Molyneaux are employed by Sirnaomics.

## ETHICS STATEMENT

The authors confirm that the ethical policies of the journal, as noted on the journal's author guidelines page, have been adhered to. No ethical approval was required, this study involved no human subjects and animal models were performed under humane protocols.

## Data Availability

The data that support the findings of this study are available from the corresponding author upon reasonable request.
